# Cytochrome P450-Induced Backbone Rearrangements in Terpene Biosynthesis of Plants

**DOI:** 10.3390/molecules30173540

**Published:** 2025-08-29

**Authors:** Maximilian Frey, Christina Marie Jochimsen, Jörg Degenhardt

**Affiliations:** 1 Department of Pharmaceutical Biology and Pharmacology, Institute of Pharmacy, Martin Luther University Halle-Wittenberg, Hoher Weg 8, 06120 Halle (Saale), Germany; 2 Department of Cell and Metabolic Biology, Leibniz Institute of Plant Biochemistry, Weinberg 3, 06120 Halle (Saale), Germany; 3 Heidelberg University, Biochemistry Center, 69120 Heidelberg, Germany

**Keywords:** P450s, terpenes, rearrangement reactions

## Abstract

Terpenes, the largest class of plant specialized products, are built from C5 building blocks via terpene synthases and oxidized by cytochrome P450 enzymes (CYPs) for structural diversity. In some cases, CYPs do not simply oxidize the terpene backbone, but induce backbone rearrangements, methyl group shifts, and carbon–carbon (C–C) scissions. Some of these reactions were characterized over 25 years ago, but most of them were reported in recent years, indicating a highly dynamic research area. These reactions are involved in mono-, sesqui-, di- and triterpene metabolism and provide key catalytic steps in the biosynthesis of plant hormones, volatiles, and defense compounds. Many commercially relevant terpenoids require such reaction steps in their biosynthesis such as triptonide (rodent pest management), secoiridoids (flavor determinants), as well as ginkgolides, cardenolides, and sesquiterpene lactones with pharmaceutical potential. Here, we provide a comprehensive overview of the underlying mechanisms.

## 1. Introduction: The Modular Biosynthesis of Terpenes

**Figure 1:**
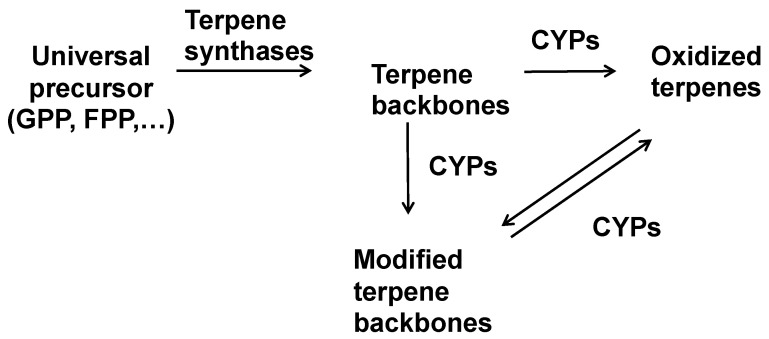
General scheme for the biosynthesis of plant terpenes.

Terpenes are the largest class of plant specialized products with an overwhelming diversity of structures [1]. This diversity is based on precursors derived from the same C5 building blocks and a modular biosynthesis of (1) generation of a terpene backbone by terpene synthases and (2) oxidation of the backbone by cytochrome P450 enzymes (CYPs) (Figure 1) [2]. Cytochrome P450 enzymes typically introduce molecular oxygen into the terpene backbone, thereby leading to the decoration of these backbones with hydroxy-, keto-, acid-, epoxy- and lactone groups [3]. Hydroxy groups often function as anchors that allow the esterification with organic acids, sugars, and other moieties. However, CYPs can carry out reactions other than these typical backbone decorations in almost all classes of terpenes (Figure 1) leading to modified terpene backbones.

**Figure 2:**
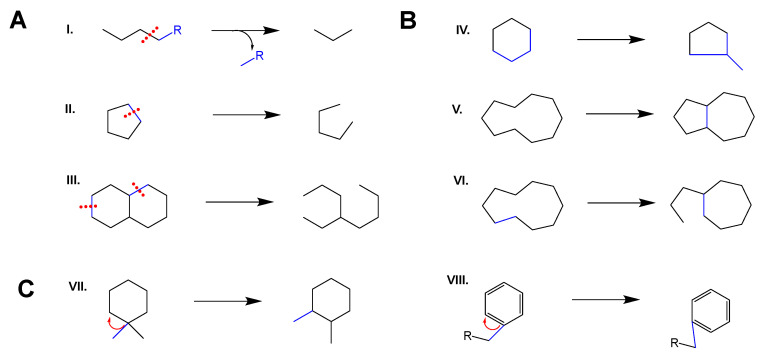
Types of CYP-induced terpene backbone modifications. (**A**) C–C scission leading to cleavage (I), or the opening of one (II) or two rings (III). (**B**) Rearrangements: ring contraction (IV), transannular cyclization without (V), and with ring opening (VI). (**C**) Group shifts: Methyl (VII) and alkyl (VIII) group shifts.

The CYP-induced modifications in plant terpene backbones include C–C cleavage (Figure 2A) resulting in the cleavage of alkyl groups (I) [4], or the opening of one (II) [5] or two (III) [6] rings. CYP-induced rearrangement reactions (Figure 2B) can include ring contraction (IV) [7,8] and transannular cyclization (V) [9], which can also occur in combination with C–C scission (VI) [10]. Most recently, group shifts were observed to be induced by CYPs (Figure 2C), such as the shift in a methyl- (VII) [11] or an alkyl group (VIII) [6] along a ring. These CYP-induced terpene backbone modifications occur on mono-, di-, sesqui-, and triterpenes, and the CYPs carrying out these reaction types are members of a diverse array of CYP families (Table 1).

## 2. C–C Scission and Ring Opening

**Figure 3:**
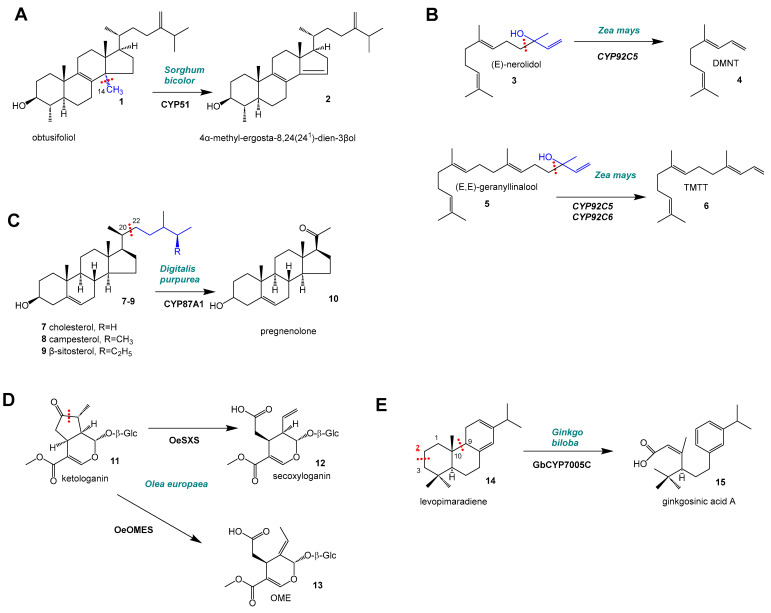
P450-induced C–C scission reactions resulting in ring opening and cleavage of side chains. (**A**) CYP51 reaction in *Sorghum bicolor*; (**B**) CYP92C5/6 reactions in *Zea mays*; (**C**) CYP87A1 reaction in *Digitalis purpurea*; (**D**) Reaction of OeSXS and OeOMES in *Olea europeana*; (**E**) CYP7005C reaction in *Ginkgo biloba*. Red, underlined: position of carbon atom that is initially oxidized. Blue: part of the molecule that is cleaved off.

An essential step in plant sterol metabolism is the demethylation (-C_1_) of obtusifoliol (**1**) to 4α-methyl-ergosta-8,24(24^1^)-dien-3β-ol (**2**) by an obtusifoliol 14α-demethylase from the CYP51G subfamily [12,13] (Figure 3A). A CYP51G enzyme from a wild potato species (*Solanum chacoense*) accepted both obtusifoliol and lanosterol as substrates for C14-demethylation [20] and the homologous enzyme from maize accepted alternative substrates such as 24α(28)-dihydro-obtusifoliol and 24,(25)-dihydro-31-norlanosterol [21]. CYPs also play a key role in the formation of homoterpenes (Figure 3B), that is often herbivore-induced: CYP92C5 in maize (*Zea mays*) can cleave off a four (-C_4_) unit from the sesquiterpene (C_15_) precursor (*E*)-nerolidol (**3**) to yield the homoterpene (*E*)-3,8-dimethyl-1,4,7-nonatriene (DMNT) (**4**) (C_11_). Also, CYP92C5 (and CYP92C6) can convert the diterpene precursor (*E*,*E*)-geranyllinalool (**5**) (C_20_) to (*E*,*E*)-4,8,12-trimethyltrideca-1,3,7,11-tetraene (TMTT) (**6**) (C_16_) [4]. The exact reaction mechanism and the fate of the cleaved carbon atoms have not been determined. In triterpene (C_30_) metabolism, there are not only examples of CYPs that induce the cleavage of a methyl group (C_1_), but also CYPs that induce the cleavage of (C_6_–C_8_) sterol side chains, which is required for the biosynthesis of cardenolides. Kunert et al. [18] characterized enzymes from the CYP87A1 subfamily that were able to convert cholesterol (**7**) (C_27_), campesterol (**8**) (C_28_), and β-sitosterol (**9**) (C_29_) to pregnenolone (**10**) (C_21_), cleaving off side chains of various lengths (C_6_–C_8_) (Figure 3C). The authors identified the corresponding CYP enzymes from the phylogenetically distant plants foxglove (*Digitalis purpurea*), treacle-mustard (*Erysimum cheiranthoides*), and giant milkweed (*Calotropis procera*). Based on comparative docking studies, the authors propose a mechanism similar to that of HsCYP11A1, the cholesterol side-chain cleavage enzyme in humans (*Homo sapiens*), in which two hydroxylation reactions of cholesterol at C20 and C22 are followed by C20–C22 cleavage of 20,22-dihydroxycholesterol [18,22].

The biosynthesis of monoterpene (C_10_) iridoids including the formation of the diffBerent stereochemical orientations that can be realized in nature is well understood in several plant species. A subgroup of these, the secoiridoids, require a ring-opening mechanism that can be carried out by various CYP enzymes. One recent example was reported by Rodriguez-Lopez et al. [5] who described the oxidative C–C cleavages of ketologanin (**11**) to form either secoxyloganin (**12**) or oleoside methyl ester (OME) (**13**) catalyzed by CYP72 enzymes (Figure 3D) in olive (*Olea europaea*). This oxidative C–C cleavage (Figure 3D) in the pathway to secoiridoids is carried out by enzymes from the CYP72A subfamily. In the early 90s, Vetter et al. (1992) [23] described the first CYP72 enzyme CYP72A1 in Madagascar periwinkle (*Catharanthus roseus*), which was assumed to play a role in iridoid metabolism, but the exact enzymatic function remained unclear. Using microsomal preparations from Japanese honeysuckle (*Lonicera japonica*), Yamamoto et al. showed that the C–C cleavage en route from loganin to secologanin is carried out by a P450 enzyme [24]. They described this enzyme as secologanin synthase (SLS) and proposed mechanisms for this P450-induced C–C cleavage. Later, Irmler et al. showed that the function of CYP72A1 was the oxidative C–C cleavage of loganin to secologanin [25]. By now, four such reactions by CYP72 enzymes have been characterized in detail in three plant species: 1. conversion of loganic acid to secologanic acid in *Camptotheca acuminata* by secologanic acid synthases (SLASs) (CYP72A565, CYP72A610) [26]; 2. conversion of loganin to secologanin in Madagascar periwinkle (*Catharanthus roseus*) by CrSLS [25]; 3. conversion of ketologanin (**11**) to either secoxyloganin (**12**); or 4. conversion of ketologanin to OME (**13**) in olive (*Olea europaea*) [5]. Interestingly, CYP enzymes from the CYP72A subfamily are involved in a wide range of terpenoid biochemical pathways, such as monoterpene (iridoids), diterpene (gibberellin), and triterpenoid (steroids, avenacin) biosynthesis. While elucidating the metabolic pathways to ginkgolides in ginkgo, Forman et al. [6] observed an intriguing CYP reaction: an 2α-hydroxylation of levopimaradiene (**14**) with a subsequent opening of ring A and B of levopimaradiene backbone ultimately yielding ginkgosinic acid A (**15**) by *Gb*CYP7005C1.

## 3. Ring Rearrangements of the Backbones (IV)–(VI)

**Figure 4:**
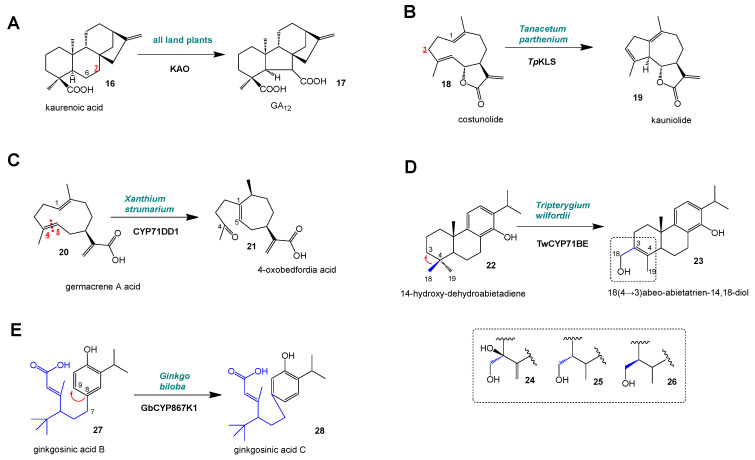
P450-induced rearrangement reactions and alkyl group shifts in plant terpene biosynthesis. (**A**) Kaurene oxidase (KAO) reaction in all land plants; (**B**) KLS reaction in *Tanacetum parthenium*; (**C**) CYP71DD1 reaction in *Xanthium strumarium*; (**D**) CYP71BE reaction in *Tripterygium wilfordii*; (**E**) CYP867K1 reaction in *Ginkgo biloba*. Red, underlined: position of carbon atom that is initially oxidized. Blue: Side chain that was shifted by the P450 reaction.

One of the most conserved terpenoid pathways in land plants is the formation of gibberellin plant hormones. A key step in this pathway is the conversion of kaurenoic acid (**16**) to GA_12_ (**17**) by kaurenoic acid oxidases (KAOs) from CYPs of the CYP88A subfamily [7,8] (Figure 4A). This three-step reaction proceeds via 7-hydroxy kaurenoic acid and 7-keto kaurenoic acid. In the final step, the formation of an acid group at C7 and the ring B contraction from C6 to C5 are catalyzed.

In a large subclass of sesquiterpenes (C_15_), the sesquiterpene lactones (STLs), CYPs contribute to the formation of specific backbones. Not all terpene structures observed in nature are the result of terpene synthase reactions. For instance, the class of STLs with >5000 compounds contains dozens of backbone types, most of which are believed to arise from a common germacrene backbone ring [27,28]. The formation of guaianolides is enabled by kauniolide synthases (KLSs) that convert costunolide (**18**) to kauniolide (**19**) in feverfew (*Tanacetum parthenium*) and chicory (*Cichorium intybus*) [9,19,29] (Figure 4B). The formation of xanthanolide STL is initiated by the conversion of germacrene A acid (**20**) to 4-oxobedfordia acid (**21**) by CYP71DD1 in *Xanthium strumarium* [10] (Figure 4C).

## 4. Methyl/Alkyl Group Shifts

In diterpene (C_20_) biosynthesis, many unusual P450 reactions are reported. Hansen et al. [11] observed a CYP-induced shift in a methyl (C_1_) group along the ring of a diterpene in the thunder god vine (*Tripterygium wilfordii*) biosynthetic pathway to triptonide. Two enzymes from the CYP71BE subfamily convert 14-hydroxy-dehydroabietadiene (**22**) to 18 (4 → 3) abeo-abietatrien-14,18-diol (**23**), shifting the C18 methyl group from C4 to C3 along the A ring (Figure 4D). These CYP reactions resulted in a variety of products with a shifted methyl group (**24**–**26**). In a later step of ginkgolide biosynthesis, ginkgosinic acid B (**27**) is converted to ginkgosinic acid C (**28**) by CYP867K1 (Figure 4E). Intriguingly, CYP867K1 shifts the branched C_11_ side chain from C8 to C9 along the C ring.

## 5. Underlying Mechanisms

### 5.1. Ring Rearrangement Mechanisms

In the biosynthesis of gibberellins (diterpenes) KAO converts ent-kaurenoic acid (**16**) to GA_12_ (**17**) in three steps without intermediate release. *En*t-Kaurenoic acid undergoes C7 hydroxylation, ring contraction, then oxidation to GA_12_ [7,8,30].

**Figure 5:**
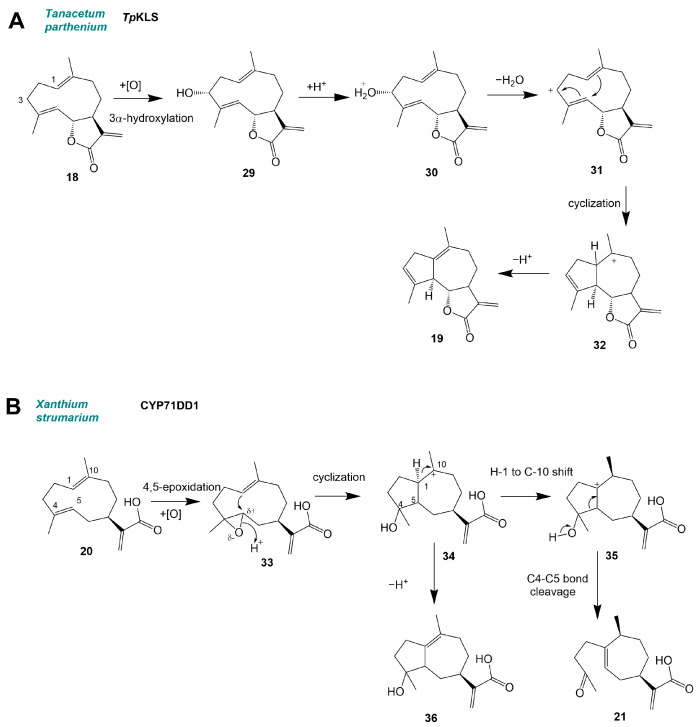
P450-induced rearrangement reactions of sesquiterpenes. (**A**) Kauniolide synthase (modified from Liu et al., 2018) and (**B**) CYP71DD1 (modified from Li et al., 2024) [9,10].

In sesquiterpene biosynthesis the germacrene backbone is prone to acid-induced rearrangements to eudesmanolides and heat-induced Cope rearrangements to elemenes [28,31], which can be artifacts of the analytical technique. However, this formation of eudesmanolide backbones after P450 oxidation was recently observed in planta [32]. Two major STL backbone types, the xanthanolides and the guaianolides, were recently shown to be derived from the common germacrenolide precursor germacrene A acid (GAA) (**20**). In the case of the guaianolide STL kauniolide a “post-lactone” pathway requires the lactone formation from GAA to costunolide (**18**), before the conversion of the backbone. Liu et al. [9] discovered that the enzyme *Tp*KLS can convert costunolide (**18**) into 3α-hydroxycostunolide (**29**), which is subsequently converted into kauniolide (**19**) (Figure 5).

In the proposed mechanism of the backbone rearrangement, the 3α-hydroxy group of this intermediate is protonated (**30**) [9]. The loss of a water molecule then leads to a C3 carbocation (**31**) that induces the ring rearrangement to (**32**). After protonation kauniolide (**19**) is formed [9]. Recently, homologous KLS enzymes belonging to the same P450 subfamily were characterized in chicory (*Cichorium intybus*), albeit with a relatively low amino acid identity [19,29]. All kauniolide synthases characterized thus far belong to the CYP71BZ subfamily. In the “pre-lactone” pathway recently described by Li et al. [10], the backbone conversion occurs before the formation of a lactone ring, challenging previous assumptions [27] about the formation of *Asteraceae* STL. In *Xanthium strumarium*, GAA is converted to 4-oxobedfordia acid (**21**) by CYP71DD1, ultimately leading to the formation of the 7,8-cis-xanthanolide STLs 8-epi-xanthatin and tomentosin [10]. Based on previously reported mechanisms for the formation of xanthanolides from the 4,5-epoxide parthenolide [33], the authors suggest a mechanism that includes oxygenation, cyclization, and C–C bond scission: In the first step, CYP71DD1 converts GAA (**8**) to 4,5-epoxy-GAA (**33**). Starting from 4,5-epoxy-GAA, a 1,10-double bond attack initiates transannular cyclization to intermediate (**34**), followed by either cleavage to (**21**) or cyclization to (**36**) [10]. A similar mechanism could be responsible for the formation of 7,8-cis lactone guaianolide STL. The CYP71DD subfamily appears to be specific to *Asteraceae* STL metabolism [28], where enzymes from this subfamily typically introduce hydroxy groups into oxidized sesquiterpene backbones such as germacrene A acid (**20**) (CYP71DD1), kauniolide (**19**) (CYP71DD5) [9], 8β-hydroxygermacrene A acid (CYP71DD6) [32], or 8-deoxylactucin [34] (CYP71DD33).

### 5.2. Cleavage Mechanisms

**Figure 6:**
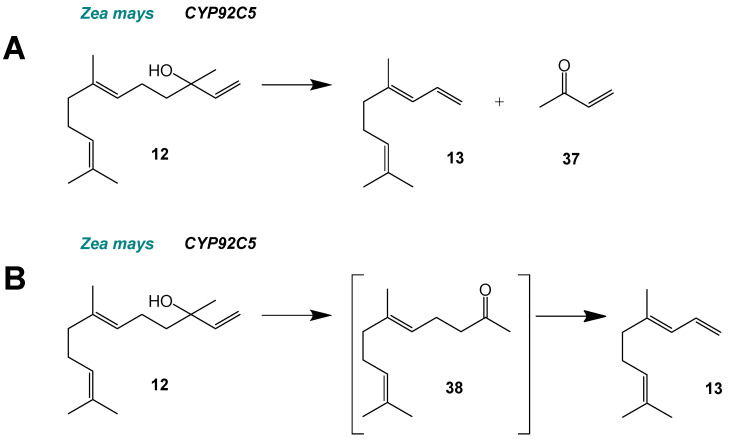
Side chain cleavage by CYP92 enzymes. (**A**) Single step fragmentation. (**B**) Two-step fragmentation proceeding via the consecutive loss of two C2 moieties. Figure modified from Lee et al. (2010) [15].

The formal by-product of the one-step oxidative degradation (Figure 6A) of (*E*)-nerolidol (**12**) to DMNT (**13**) is but-3-ene-2-one (methyl vinyl ketone) (**37**) (C_4_), but it has not been detected as a by-product of homoterpene formation [35,36]. An alternative two-step reaction mechanism has been postulated, that proceeds via the ketone intermediates geranylacteone (**38**) and farnesylacetone, respectively, (Figure 6B) [36]. In this mechanism, two carbon (C_2_) units would be cleaved, but experimental evidence for it remains inconclusive. Small amounts of geranylacetone have been detected in DMNT-containing volatile blends [37], and geranylacetone was converted in feeding experiments with lima bean (*Phaseolus lunatus*) and purple magnolia (*Magnolia liliiflora nigra*) [36]. In contrast, the ketone intermediates were not detected in assays with the thale cress (*Arabidopsis thaliana*) CYP82G1 and they were shown to be inefficient substrates for the conversion to homoterpenes catalyzed by this enzyme [15]. Even though the reaction mechanism requires further investigation, some mechanistic aspects have already been elucidated, namely, that it proceeds via *syn*-elimination of the oxygen-carrying group with a hydrogen atom of the allylic β-carbon [38]. In rice, OsCYP92C21 produces both DMNT and TMTT [39]. This reaction can also be carried out by enzymes from the CYP82 family: CYP82D in tea (*Camellia sinensis*) [14], CYP82G in thale cress (*A. thaliana*) [15], and CYP82L in cotton (*Gossypium hirsutum*) [16] and orange (*Citrus sinensis*) [17].

Rodriguez-Lopez et al. [5] have suggested reaction mechanisms for the CYP-catalyzed oxidative cleavages en route to secoiridoids in olive (Figure S1). *Cr*SLS, which oxidizes loganin (**39**) via (**40**,**41**) to secologanin (**42**) (Figure S1A), follows a similar mechanism as OeSXS, which oxidizes ketologanin (**5**) via (**43**,**44**) to secoxyloganin (**6**) (Figure S1B) while *Oe*OMES converts ketologanin (**5**) to OME (**7**) via (**45**,**46**) (Figure S1C). The authors speculate that the stereochemistry of the hydroxyl group in position 7 of loganin determines the orientation of the substrate in the *Cr*SLS binding site, such that the hydrogen of C10 can be abstracted by the iron cofactor, ultimately leading to secologanin [24] (Figure S1A).

*Gb*CYP7005C1/7005C3 cleave two C–C bonds in levopimaradiene (**14**), with a radical shift from C3 to C1, preserving the tert-butyl group (Figure S1D) via intermediates (**47**–**49**). As proposed by Schwarz and Arigoni [40], a hydrogen shift enables oxygen rebound and subsequent rearrangement via aromatization, dehydration, and heterolytic cleavage (Figure S1C) via intermediates (**50**,**51**) to ginkgosinic acid A (**15**) [6].

### 5.3. Group Shift Mechanisms

The C7–C8 to C7–C9 shift from ginkgosinic acid B (**27**) to ginkgosinic acid C (**28**) may proceed via a dienone-phenol NIH rearrangement via intermediate (**52**) (Figure S2A). Alternatively, epoxidation with acid-catalyzed opening and alkyl migration via intermediates (**53**–**55**) yields ginkgosinoic acid C. In the conversion of 14-hydroxy-dehydroabietadiene (**22**) to multiple products (**23**–**26**; **56**–**58**) (Figure S2B), a hypothetical Wagner–Meerwein rearrangement explains the C18 to C3 methyl shift in the abietane backbone [11].

## 6. Conclusions and Perspectives

In recent years, there has been a growing number of reports of unusual P450 reactions. These observations may pave the way forward for the elucidation of metabolic pathways with so far elusive mechanisms. One example is the furan ring formation in cafestol biosynthesis, which requires a methyl group shift in the kaurene backbone, similar to what has been observed in triptolide biosynthesis. It would be very interesting to screen P450s with non-canonical reactions in high-throughput assays for alternative substrates to exploit their unique biosynthetic activity for other chemical reactions. Site-directed mutagenesis studies guided by substrate docking as well as feeding assays with hypothesized intermediates can provide a more detailed understanding of the sequence of reactions carried out by these P450s. With the advancements in genome sequencing, high-throughput testing, as well as metabolomics and transcriptomics on a cellular level, we expect the elucidation of additional P450-induced rearrangement reactions in the future.

## Figures and Tables

**Table 1:** Reaction types and CYP subfamilies.

Type	Reactions (References)	P450 Subfamily	Enzymes	Number *	Terpene Substrate
I	C–C scission, cleaving of C1 unit, demethylation of obtusifoliol ([12,13])	CYP51G		3	Triterpene
I	C–C scission, cleave of a four (-C_4_) unit from sesquiterpene or diterpene precursors to produce C11- or C16-homoterpenes ([4,14,15,16,17])	CYP92C	CYP92C5,6	2	Sesqui-/Diterpene
		CYP82D/G/L		4	
I	C–C scission, cleaving of side chains of various lengths (C_6_–C_8_) to produce cardenolides via pregnenolone ([18])			3	Triterpene
II	C–C scission in the formation of secoiridoids ([5])	CYP72A	SLAS, SLS, SXS, OMES	4	Monoterpene
III	C–C scission, ring opening of ring A and ring B in levopimaradiene ([6])	CYP7005C	CYP7005C1/C3	2	Diterpene
IV	Ring contraction of kaurenoic acid B ring in the gibberellin biosynthesis ([8])	CYP88A	KAO	>10	Diterpene
V	Ring rearrangement, transannular cyclization, formation of 6,7-trans guaianolides ([9,19])	CYP71BZ	KLS	4	Sesquiterpene
VI	Ring rearrangement, transannular cyclization, formation of 7,8-trans xanthanolides ([10])	CYP71DD	CYP72DD1	1	Sesquiterpene
VII	Methyl group shift, 18 (4 → 3), via Wagner–Meerwein rearrangement ([11])	CYP71BE		2	Diterpene
VIII	Alkyl group shift along aromatic ring from C8 to C9 of ginkgosinic acid B ([6])	CYP867K	GbCYP867K1	1	Diterpene

Note: In almost all cases, the CYP subfamilies include enzymes with various other substrate and reaction types. * Number of enzymes catalyzing this reaction.

## Data Availability

No new data were created or analyzed in this study. Data sharing is not applicable to this article.

## Supplementary Materials

The following supporting information can be downloaded at https://www.mdpi.com/article/10.3390/molecules30173540/s1, Figure S1: Pathways for C–C scission by CYP enzymes; Figure S2: CYP-induced group shifts.
