# Supplementary material for: Cytochrome P450-Induced Backbone Rearrangements in Terpene Biosynthesis of Plants
Source: Molecules. 2025 Aug 29;30(17):3540. doi: 10.3390/molecules30173540 (PMC12430055; doi:10.3390/molecules30173540)
Supplement: Supplementary file 1 [file molecules-30-03540-s001.zip › molecules-3727033-supplementary.pdf]

Supporting Material

Supporting Figures

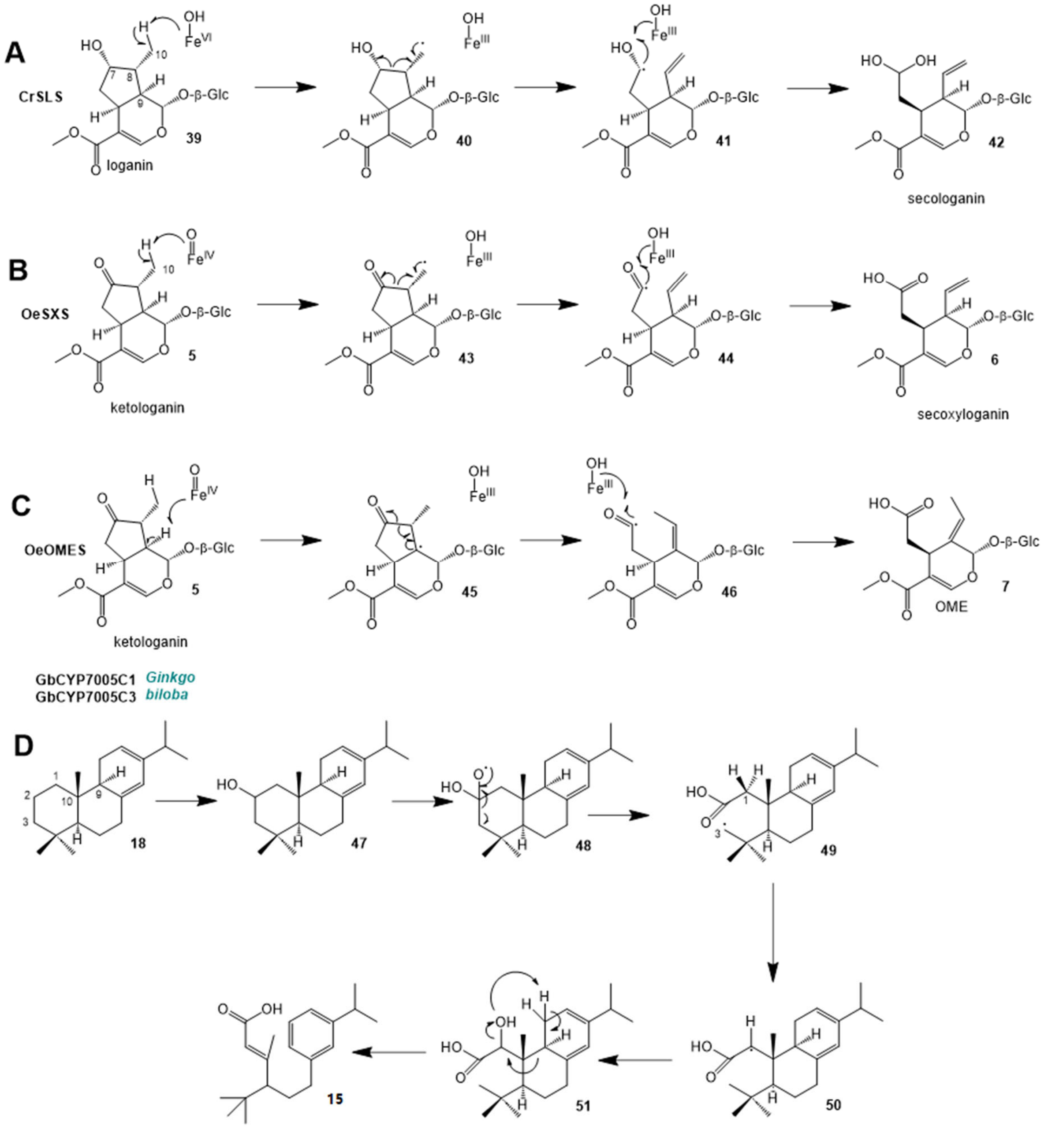

Figure S1: Pathways for C–C scission by CYP enzymes: Conversion of A) loganin to secologanin by *Cr*SLS. B) ketologanin conversion to secoxyloganin by *Oe*SXS and C) to oleoside methyl ester (OME) via *Oe*OMES. D) Conversion of levopimaradiene to ginkgosinic acid A. Figure S1A and S1B modified from Rodriguez-Lopez et al. 2021; Figure S1C modified from Forman et al. 2022. [1, 2]

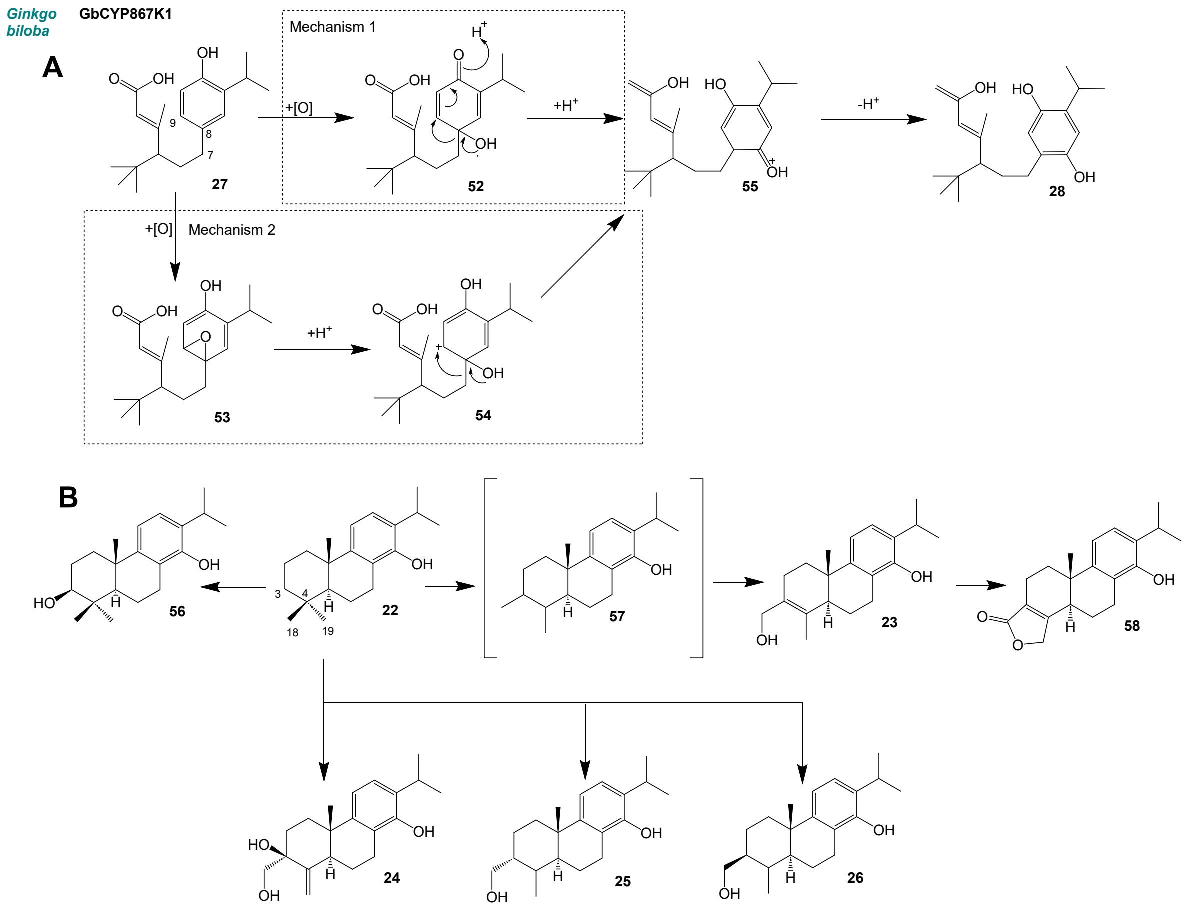

Figure S2: CYP-induced group shifts in the reactions from A) ginkgosinic acid B to ginkgosinic acid C and B) from 14-hydroxy-dehydroabietadiene to various products. Figure 2A modified from Forman et al. 2022, Figure 2B modified from Hansen et al. 2022 [2, 3]

1. C. E. Rodríguez-López, B. Hong, C. Paetz, Y. Nakamura, K. Koudounas, V. Passeri, L. Baldoni, F. Alagna, O. Calderini and S. E. O'Connor, *New Phytologist*, 2021, **229**, 2288-2301.
2. V. Forman, D. Luo, F. Geu-Flores, R. Lemcke, D. R. Nelson, S. C. Kampranis, D. Staerk, B. L. Møller and I. Pateraki, *Nature Communications*, 2022, **13**, 5143.
3. N. L. Hansen, L. Kjaerulff, Q. K. Heck, V. Forman, D. Staerk, B. L. Møller and J. Andersen-Ranberg, *Nature Communications*, 2022, **13**, 5011.
